# Investigating the Efficacy and Cost-Effectiveness of Technology-Delivered Personalized Feedback on Dietary Patterns in Young Australian Adults in the Advice, Ideas, and Motivation for My Eating (Aim4Me) Study: Protocol for a Randomized Controlled Trial

**DOI:** 10.2196/15999

**Published:** 2020-05-22

**Authors:** Rebecca L Haslam, Kristine Pezdirc, Helen Truby, John Attia, Melinda Hutchesson, Tracy Burrows, Robin Callister, Leanne Hides, Billie Bonevski, Deborah A Kerr, David Lubans, Sharon Kirkpatrick, Megan Rollo, Tracy McCaffrey, Clare E Collins

**Affiliations:** 1 Priority Research Centre for Physical Activity and Nutrition School of Health Sciences University of Newcastle Callaghan Australia; 2 Faculty of Health and Medicine University of Newcastle North Sydney Australia; 3 Department of Nutrition, Dietetics and Food Monash University Clayton Australia; 4 School of Medicine and Public Health Faculty of Health and Medicine University of Newcastle Callaghan Australia; 5 Priority Research Centre for Physical Activity and Nutrition School of Biomedical Sciences and Pharmacy University of Newcastle Callaghan Australia; 6 School of Psychology University of Queensland Brisbane Australia; 7 School of Public Health Faculty of Health Sciences Curtin Institute of Technology Perth Australia; 8 Priority Research Centre for Physical Activity and Nutrition School of Education University of Newcastle Callaghan Australia; 9 School of Public Health and Health Systems University of Waterloo Waterloo, ON Canada

**Keywords:** young adults, web-based, dietary feedback, nutrition, eHealth, diet

## Abstract

**Background:**

Web-based health interventions may be easier to access and time efficient relative to face-to-face interventions and therefore may be the most appropriate mode to engage young adults.

**Objective:**

This study aims to investigate the impact of 3 different levels of personalized web-based dietary feedback and support on changes in diet quality.

**Methods:**

The Advice, Ideas, and Motivation for My Eating (Aim4Me) study is a 12-month assessor-blinded, parallel-group randomized controlled trial evaluating the impact of 3 levels of web-based feedback on diet quality, measured using the Australian Recommended Food Score (ARFS). Participants (N=2570) will primarily be recruited via web-based methods and randomized to 1 of 3 groups. Group 1 (control) will receive the Healthy Eating Quiz, a web-based dietary assessment tool that generates a *brief* feedback report on diet quality. Individuals randomized to this group can use the *brief* feedback report to make positive dietary changes. Group 2 will receive the Australian Eating Survey, a web-based dietary assessment tool that generates a *comprehensive* feedback report on diet quality as well as macro- and micronutrient intake. Group 2 will use the *comprehensive* feedback report to assist in making positive dietary changes. They will also have access to the Aim4Me website with resources on healthy eating and tools to set goals and self-monitor progress. Group 3 will receive the same intervention as Group 2 (ie, the *comprehensive* feedback report) in addition to a tailored 30-min video consultation with an accredited practicing dietitian who will use the *comprehensive* feedback report to assist individuals in making positive dietary changes. The self-determination theory was used as the framework for selecting appropriate website features, including goal setting and self-monitoring. The primary outcome measure is change in diet quality. The completion of questionnaires at baseline and 3, 6, and 12 months will be incentivized with a monetary prize draw.

**Results:**

As of December 2019, 1277 participants have been randomized.

**Conclusions:**

The web-based delivery of nutrition interventions has the potential to improve dietary intake of young adults. However, the level of support required to improve intake is unknown.

**Trial Registration:**

Australian New Zealand Clinical Trials Registry ACTRN12618000325202; https://www.anzctr.org.au/Trial/Registration/TrialReview.aspx?id=374420

**International Registered Report Identifier (IRRID):**

DERR1-10.2196/15999

## Introduction

### Background

In Australia, young adults (aged 18-24 years) are gaining weight and at a faster rate than any other adult age group [1,2], with 31.5% [3] affected by overweight or obesity. Studies report weight gain of around 0.5 kg to 1 kg (1-2 lbs) per year over a 5- to 10-year period in young adults [4-6]. Becoming overweight or obese at a young age increases the risk of noncommunicable chronic diseases, including metabolic syndrome, type 2 diabetes, cardiovascular disease, and specific cancers [7].

Diet quality is currently poor among young adults [8], with the high consumption of sugar-sweetened beverages (SSBs) and low intake of fruit and vegetables [3]. Discretionary foods (predominantly SSBs, alcohol, and takeaway and convenience foods) account for over one-third of total energy intake in Australia [9]. Globally, similar patterns of dietary intake have been observed, with young adults having the lowest diet quality [10]. Dietary patterns also differ by income status and ethnicity across regions [11], but when comparing the diet quality of low- and high-income countries, young adults still have the poorest diet across income levels [11]. Poorer diet quality is linked to poor physical and mental health [12-14], and considering that the dietary habits of young adults have been shown to track throughout life when disease risk is higher [15], intervening during young adulthood is crucial [16].

Young adults are faced with multiple life-stage challenges, including moving out of or away from home, commencing study or employment, developing new social interactions or cohabitations, and increased independence and financial responsibilities [2]. These changes can interfere with the adoption of healthy eating behaviors. Young adults reported the following key barriers to eating healthy: lack of time (because of balancing work, study, and a social life); lack of skills and knowledge to plan, shop, prepare, and cook healthy foods; relative low cost and availability of less healthy foods; peer influences and lack of motivation to eat healthy; and competing priorities [2,17,18]. Strategies to help overcome these barriers are required, and because of the unique characteristics of this group and the challenge with reaching and engaging them in health behavior change, an appropriate set of strategies for this age group needs to be selected. The self-determination theory (SDT) supports self-directed motivation by satisfying an individual’s need for autonomy, perceived competence, and relatedness and focuses on the extent to which behaviors are self-initiated (autonomous) versus influenced by external factors (external motivators) [19]. At the center of the Behavior Change Wheel framework is a system comprising 3 key factors that influence behavior change: capability, opportunity, and motivation (COM-B) [20]. It provides a framework for selecting appropriate intervention strategies, such as goal setting, tracking, and action planning, which are essential for building long-term positive behavior changes in young adults. A recent review reported that the most frequently used behavior change techniques for improving the dietary intake of young adults included goal setting and feedback on behavior [21]. Goal setting includes setting or agreeing on a goal, defined in terms of the behavior to be achieved (eg, *increase serves of fruit by one serve per day*)*.* Feedback on behavior is where goals and behaviors are monitored and informative or evaluative feedback is provided on the performance of the behavior (*eg, frequency or quantity of intake of fruit*) [22]. Interventions including behavior change techniques have been shown to be more effective at improving dietary intake compared with those without [21].

Beyond the selection of appropriate strategies, interventions targeting young adults need to consider the ideal mode of delivery for optimal engagement. The uptake of web-based technologies to support health is still increasing in young adults, and web-based technologies continue to evolve to meet this demand. Websites offer a platform for information delivery via various modes, including written, audio, and video, and advances in technology allow web-based programs to be accessed via mobile devices, such as smartphones [23]. Additional benefits of web-based interventions include greater reach in terms of geographical location and population groups and the ability to maximize the collection of complete data [23].

The Advice, Ideas, and Motivation for My Eating (Aim4Me) study aims to recruit young adults and provide nutrition interventions with varying levels of feedback, nutrition education, goal setting and tracking, and interaction with a dietitian in a web-based environment. The extent to which these types of interventions can successfully recruit, engage, and effect positive dietary change in this population has not been investigated.

### Aim

Thus, the primary aim is to investigate the impact of 3 levels of personalized dietary feedback and support on changes in diet quality, as measured by the Australian Recommended Food Score (ARFS). The secondary aim is to investigate intervention reach, participant engagement, retention, satisfaction, and cost-effectiveness.

## Methods

### Study Design

Aim4Me is a 12-month assessor-blinded, parallel-group randomized controlled trial assessing the impact of varying levels of web-based feedback on diet quality. The study is approved by the University of Newcastle Human Research Ethics Committee (H-2017-0087). This study was prospectively registered with the Australian New Zealand Clinical Trials Registry and is consistent with the Consolidated Standards of Reporting Trials guidelines (ACTRN #12618000325202) [23]. The study was performed in accordance with the Declaration of Helsinki. All patients enrolled in the study provided written consent.

**Figure 1:**
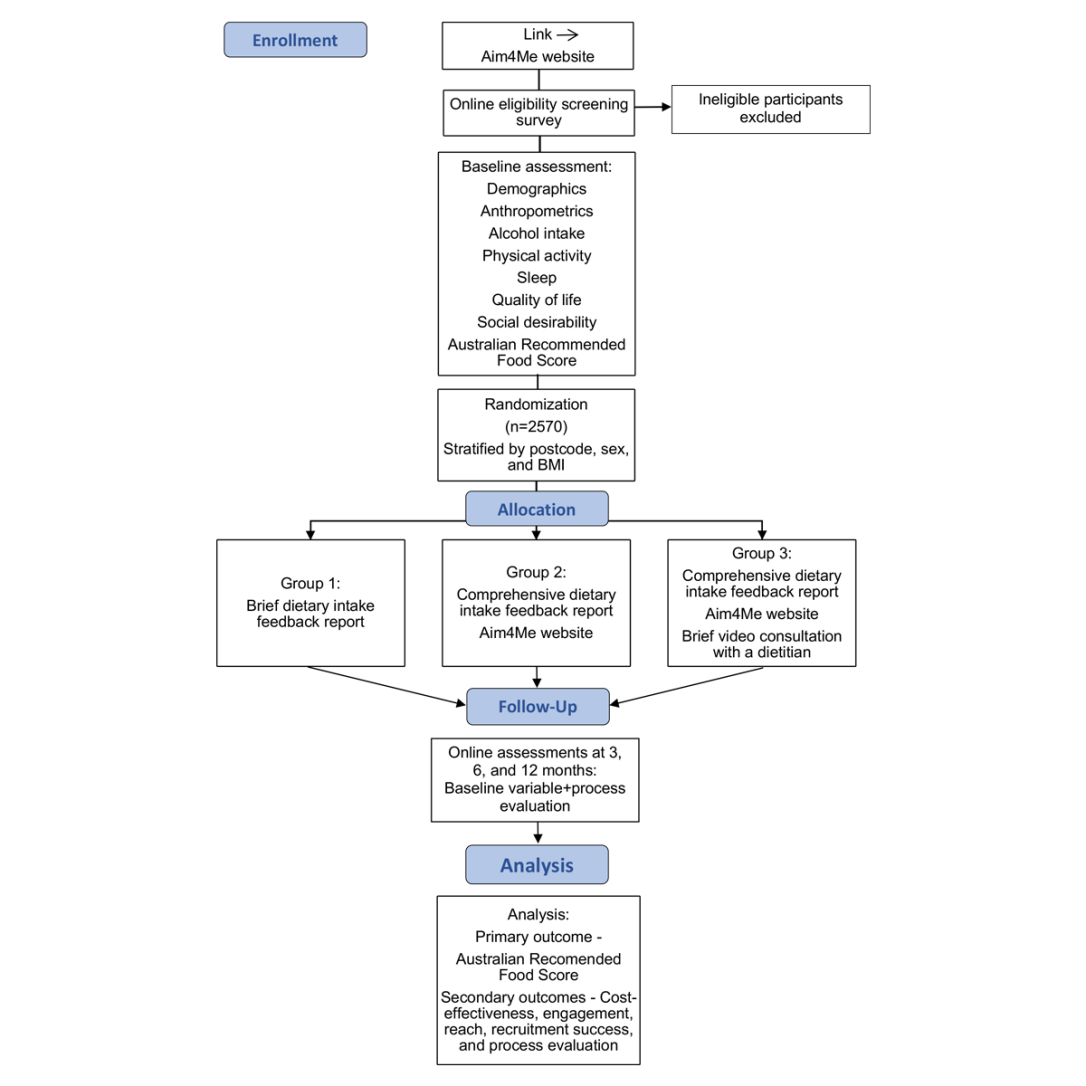
Study flowchart. Aim4me: Advice, Ideas, and Motivation for My Eating.

Participants (N=2570) will be recruited nationally across Australia. After informed consent and baseline data are collected, eligible participants will be randomized to 1 of 3 groups (Figure 1). Group 1 will receive a *brief* feedback report on their current dietary intake, whereas groups 2 and 3 will receive a *comprehensive* personalized feedback report on their usual intake as well as get access to the study website. Group 3 will also be offered a 30-min video consultation with a dietitian.

### Participants

#### Recruitment and Setting

A total of 2570 young adults (aged 18 to 24 years) will be recruited across Australia using multiple strategies. Given the target age group, a large focus of the recruitment is on using social media platforms, including Facebook, Instagram, and Twitter. An Aim4Me account will be created for each of these platforms, and specific posts suitable to each of the platforms will be developed. Paid Facebook advertising will also occur [24], delivered by institutional marketing and media teams. Study information and links to social media accounts will be shared on the websites of universities and research institutes. Flyers will be distributed around universities and to professional organizations and communities aimed at young adults and student associations, with requests that they advertise and share study information on their social media platforms, websites, or email lists. In addition, study information will be disseminated using local and national media releases via printed newspapers, magazines, and radio stations. A snowball method, in which individuals who visit the Aim4Me website will be able to share the link with their colleagues and friends via Facebook, Twitter, or email, will also be used. Finally, email campaigns will be distributed to contacts who have previously signed up to receive notifications of nutrition-related research studies through members of the research team.

Through each strategy, interested individuals will be directed to a study website to access information about what participation entails, check their eligibility, and provide informed consent, if eligible and interested.

#### Screening and Baseline Assessments

Potential participants will complete a web-based screening survey to determine whether they meet the eligibility criteria. Once deemed eligible, they will register their contact details and provide informed consent to participate in the research. A confirmation email will be sent to the address specified by the participant with their log-in details and password, allowing them to access the participant portion of the study website and commence the baseline assessment questionnaires. Email and text reminders will be sent to registered participants on an automated schedule if they have not completed the baseline assessments post screening and consent.

#### Inclusion Criteria

Eligibility criteria include being aged 18 to 24 years, residing in Australia, computer/internet access, self-reported BMI ≥18.5 kg/m^2^, not pregnant or planning pregnancy in the next year, no medical conditions, and no diagnosis of current or previous eating disorder. Participants with medical conditions, such as type 1 diabetes or Crohn’s disease, who require specific nutrition advice, will be advised to visit their general practitioner to obtain medical clearance before participating in the study.

### Randomization

Eligible participants will be randomly allocated (1:1:1) to a control group or 1 of 2 intervention groups. Randomization will occur in permuted blocks using random blocks of varying size and be stratified by postcode location (using the Monash Modified Model) [25], sex, and BMI (18.5-24.9 kg/m^2^ vs ≥25 kg/m^2^). Randomization will be coded by an independent statistician who will provide the coding to the software developers to program the web-based environment. The research team will remain blinded to the randomization code.

### Interventions

The intervention components are based on SDT, which focuses on the extent to which behaviors are self-initiated (autonomous) versus influenced by external factors (external motivators) [19]. SDT supports self-directed motivation by satisfying an individual’s need for autonomy, perceived competence, and relatedness. Autonomy will be satisfied by providing opportunities for participants to choose their own goals, reflect on progress and revise goals, access a range of resources that they self-select, and choose their level of engagement with the website. For Group 3, engagement with the dietitian will reinforce the importance of nutrition relative to personal motivators. The graphic design of the Aim4Me website and resource materials related to the motivators for, and barriers to, healthy eating among young adults. Website images, wording, and content have been selected based on feedback from this age group. In groups 2 and 3, perceived competence will be addressed by the self-development of personalized goals and regular self-monitoring of progress toward these goals to support progressive and small behavior change toward healthy eating.

#### Healthy Eating Quiz Brief Dietary Intake Feedback Report (Group 1)

The Healthy Eating Quiz (HEQ) [26] will be available via a link on the Aim4Me dashboard, which will direct Group 1 to this intervention component outside of the website. The HEQ is a 5-min web-based dietary assessment tool that provides *brief* general feedback on current eating patterns and diet quality using the ARFS [27,28]. Group 1 will have access to the brief report to identify the key areas for improving diet quality (eg, increase the variety of vegetable intake). These participants will have access to the HEQ throughout the study and will be prompted to complete it at baseline and at 3, 6, and 12 months.

#### Australian Eating Survey Comprehensive Feedback Report (Groups 2 and 3)

The Australian Eating Survey (AES) is an automated web-based food frequency questionnaire (FFQ) that assesses usual dietary intake in adults [29]. Following completion of the AES, participants randomized to groups 2 and 3 will be provided with a real-time *comprehensive* personalized feedback report that compares usual dietary intake with Australian dietary recommendations (percent energy from 5 core healthy food groups and 10 energy-dense, nutrient-poor food groups) and nutrient reference value targets (percent energy from protein, fat, saturated fat, carbohydrate, daily grams of fiber, 7 minerals, and 5 vitamins) [30], based on age and sex. The report provides feedback on diet quality, giving a total diet quality score and scores for individual food groups. Participants will be encouraged to set goals around improving diet quality. Participants in Group 2 will receive the report but no further support on the interpretation of the report or how to use the report to set goals. Group 3 will be offered additional support in the form of a video consult with an accredited practicing dietitian (APD), which will focus on using the diet quality results from the AES report to set specific goals around improving diet quality. The control group will also complete the AES to allow measurement of change in the primary outcome (see the Primary Outcome Measures section) but will not receive the AES personalized feedback report.

#### Advice, Ideas, and Motivation for My Eating Website

Participants in groups 2 and 3 will have access to the Aim4Me website for 12 months (Textbox 1). Images of the web interface are provided in Multimedia Appendix 1.

**Description of the components of Advice, Ideas, and Motivation for My Eating website.**

Personalized dietary feedback

- An automated (computer-generated) personalized feedback report on dietary intake will be available to access through the website
- The feedback report will be provided at baseline and at 3, 6, and 12 months if the appropriate dietary assessment tool is completed. This will allow the individual to compare their previous reports and self-assess change

Healthy eating resource materials

- A web-based resource library of evidence-based materials will include links to apps, articles, fact sheets, recipes and information related to healthy eating, and targeting motivators and barriers to behavior change expressed by young males and females, for example, in relation to the key barriers of cost and time, the website will include resource tips on eating healthy on a budget, quick and easy meals, and budget recipes
- Other accessible content will include *Theme of the month*, which provides educational information on a new topic each month (eg, Love your Heart is May content); *Food*, which provides short snippets of information on specific food groups, for example, how to eat more fruits and vegetables; and *Explore*, which contains other useful information such as cooking tips, app suggestions, and recipes

Goal setting

- Setting dietary goals—Participants set short-term goals based on feedback from their personalized dietary report and can either self-select from predetermined generic goals that have been developed to target each of the food groups or write their own goal
- The food groups include vegetables and salad, fruit, dairy, breads and cereals, meat and alternatives, alcoholic beverages, fatty meats, sweetened drinks, packaged snacks, confectionary, baked sweet products, fried and takeaway food, and spreads and sauces
- The listed generic goals have been designed as specific-measurable-achievable-realistic-timely goals
- They can select up to 3 goals to focus on at any one time
- At 3 and 6 months, they will be prompted by email and/or text message to revise and update their goals after they have received their personalized feedback report for intake over the preceding 3 months

Self-monitoring

- Monitoring of dietary goals—Participants will be prompted by email and text message to self-monitor their goals by going to their dashboard
- Using a 5-point scale from *very poor* to *very good*, participants will be asked to reflect on how well they did in achieving their goals and how important their goal is to them (*very important* to *not important*)
- On the basis of their responses, they will be provided with generic feedback, which will either direct them to update their goals or provide them with information that will support them in achieving their goals

#### Video Consultation With a Dietitian (Group 3 Only)

Participants randomized to Group 3 will be encouraged to book one web-based, personalized 30-min video consultation with an APD, within 14 days of enrolling in the study. Participants will be prompted via an automated email to book their appointment on receiving their personalized feedback report. This structured consultation session will entail a review of the goals the participant has set based on the personalized feedback report from the AES and assistance in setting personalized strategies to overcome self-identified barriers to healthy eating. The resources used to streamline the personalization of the session include a brief self-administered Personalized Nutrition Questionnaire (PNQ) [31] and a Personalized Nutrition Toolbox (PNT) of resources used by the dietitian to support intervention strategies tailored to the characteristics of the population of interest. The PNQ draws upon the Behavior Change Wheel theory, which comprises the COM-B system [20]. In completing the PNQ, participants will be asked to self-identify and prioritize 18 factors (capability=7, opportunity=5, and motivation=6) that they perceive to affect their ability to achieve healthy eating. The PNT is a dietitian resource that consists of intervention strategies mapped out to each factor of the PNQ and the behavior change techniques required to deliver the intervention functions. The dietitian uses the individual’s PNQ responses to guide the selection of interventions from the PNT and personalize associated strategies to address individual goals. Each dietitian was trained in the consultation protocol to ensure consistency in consultation delivery.

### Outcome Measures

Outcome measures will be completed via the Aim4Me website at baseline and at 3, 6, and 12 months, along with a process evaluation (Multimedia Appendix 2). In addition to text message and email reminders, participants may also receive a follow-up phone call to prompt the completion of questionnaires at follow-up time points.

#### Primary Outcome Measures

##### Diet Quality

Diet quality will be measured using a validated brief diet quality index, the ARFS [27,29]. The ARFS uses a subset of 70 questions related to core nutrient-dense foods recommended in the Australian Dietary Guidelines [32]. The ARFS score is calculated by summing the points within 8 subscales, with 20 questions related to vegetable intake, 12 related to fruit, 13 related to protein foods (7 to meat and 6 to vegetarian sources of protein), 12 related to breads/cereals, 10 related to dairy foods, 1 related to water, and 2 related to spreads/sauces. The total score ranges from 0 to a maximum of 73 points [33].

#### Secondary Outcome Measures

##### Dietary Intake

###### Australian Eating Survey

Nutrient intake will also be assessed using the AES. The AES is a 120-item semiquantitative FFQ, which has been validated in adults, children, and adolescents [29,34]. The frequency of food consumption for the previous 3 or 6 months (as specified) is self-reported, using options ranging from *never* to *4 or more times per day* for foods and *7 or more glasses per day* for beverages. In total, 19 questions are related to vegetables and 11 are related to fruit, with separate questions about seasonality, total daily number of fruit and vegetable serves, bread and cereals, dairy products, eggs, fat spreads, beverages, snack foods, and discretionary items. An additional 12 questions are related to food behaviors, such as the frequency of consuming takeaway foods and eating while watching TV. Nutrient intakes are computed using the Australian food composition database to generate individual mean daily macro- and micronutrient intake using food portion sizes derived from the Australian Bureau of Statistics data [29].

##### Alcohol Intake

Alcohol consumption will be reported using the validated 3-item Alcohol Use Disorders Identification Test-Consumption to assess usual weekly alcohol consumption in grams [35].

##### Weight and Height/BMI

Weight and height will be self-reported as part of the web-based assessment questionnaire and BMI calculated (kg/m^2^). Web-based self-reported height and weight have been shown to be relatively valid in relation to the measured height and weight [36].

##### Quality of Life

Quality of life will be assessed using the 6-dimensional Assessment of Quality of Life scale (AQoL-6D), which examines 20 items across 6 domains of independent living, relationships, mental health, coping, pain, and senses and provides utility scores that can be used in economic evaluations [37].

##### Self-Determination Factors

Self-determination constructs, including dietary self-regulation, habit automaticity, perceived competence, and social support related to healthy eating, will be measured. The Regulation of Eating Behaviors Scale will be used to assess motivational orientation toward regulating diet and reasons across 6 regulatory styles, with participants asked to what extent each item corresponds to a reason for regulation using a 7-point Likert scale [38]. The Self-Report Behavioral Automaticity Index measures a 4-item change in habitual behavioral patterns with regard to learned, automatic responses to situational cues [39]. The Perceived Competence Scale is a 4-item questionnaire assessing the degree to which participants feel confident about being able to make, maintain, or change participation in healthy eating [40]. Social support from family, friends, partners, or significant others will be measured using the 12-item Multidimensional Scale of Perceived Social Support questionnaire [41].

#### Covariates

##### Sociodemographic Characteristics

Participants will be asked questions about their age, sex, postcode, ethnicity, education level, employment and income status, relationship status, living arrangements, and food security at 3, 6, and 12 months.

##### Self-Reported Physical Activity, Sitting Time, and Sleep

Physical activity (PA) and sitting time during the previous 7 days will be self-reported using the 7-item Godin Leisure-Time Exercise (frequency and duration of time in light/moderate/vigorous PA) [42] and the Marshall Sitting Time Questionnaire, respectively [43]. The Epworth Sleepiness Scale will be used to measure self-reported sleep [44]. This 8-item scale measures the general level of daytime sleepiness or average sleep propensity in daily life [44].

##### Smoking

Two items will be used to measure smoking: (1) Do you currently smoke any tobacco products? and (2) Would you have smoked 100 or more cigarettes or equivalent tobacco in your life? [45]. Moreover, 7-day abstinence will be measured at follow-up: “Have you smoked at least part of a cigarette in the last 7 days?” [46].

##### Depression, Anxiety, and Stress

Participants will complete the 21-item Depression, Anxiety and Stress Scale, which is a set of 3 self-report scales designed to measure the emotional states of depression, anxiety, and stress [47]. Each of the 21 items in the scale asks participants to report how much each item applied to them over the previous week using 4 responses (*never*, *sometimes*, *often*, or *almost always*) [47]. For example, “I found it hard to wind down.”

##### Social Desirability and Approval

Social desirability and approval have emerged as sources of bias in self-reporting of dietary intake [48]. Social desirability will be measured using the 13-item Marlowe-Crowne Social Desirability Scale [49] in which participants are prompted to answer true or false to a number of statements concerning personal attitudes and traits [49]. The Martin-Larsen Approval Motivation Scale, a 20-item 5-point Likert scale, will be used to measure social approval [50].

##### Social Influences on Food Intake

The Social Eating Scale will be used to measure influences such as culture, family, or peers on food intake, requiring participants to select the appropriate response from 6 questions using a 5-point Likert scale [51].

#### Economic Measures

There is no basis for anticipating that health service utilization will vary between trial arms as a result of the interventions. As a consequence, health service engagement is assumed to be randomized, and the requirement to collect health service engagement or medication use is excluded. The additional costs relating to the intervention and implementation of the intervention, including materials, labor, and other expenditures, will be collected through project management and project team records. The economic analysis will use either the change in ARFS or AQoL-6D as the outcome of interest. If the change in either is not statistically significant, all consequence measures, primary and secondary, will be reported alongside the cost estimates.

#### Engagement

Engagement will be measured using usage statistics captured by the website. The outcomes will include completion of the HEQ (control group only), the number of log-ins to the website, clicks on resources and links, views of personalized dietary feedback, and views and completion of goal setting and tracking (intervention groups 1 and 2). In addition, for intervention Group 2, engagement will be measured by attendance at the brief video consultation.

#### Reach and Recruitment Success

The number of people who engage with the various online recruitment strategies will be measured by using Bitly links [52], and the number of people who access and engage with the website will be measured by using Google Analytics. Bitly links allow the creation of customized URLs, which track back to the Aim4Me website and allow tracking of engagement with various strategies. Recruitment success will be measured by the time to recruit per strata, representativeness of the sample, number of people who expressed interest, percentage of eligible participants, and the number of those who consented. As part of the baseline assessment questionnaires, eligible participants will be asked how they found out about the study to capture which recruitment strategies were most successful.

#### Retention

Retention will be assessed as the proportion of participants who complete the AES at 3, 6, and 12 months.

#### Satisfaction

Self-reported satisfaction with study components will be evaluated at 3, 6, and 12 months using a process evaluation developed by the research team. Group 1 will be asked about their satisfaction with the HEQ if it was completed. For groups 2 and 3, questions will cover the personalized dietary feedback report, resources on the Aim4Me website, goal setting and tracking, and overall intervention satisfaction. Intervention Group 3 will also self-report satisfaction with the video consultation with the APD.

#### Scheduled Reminders and Prize Draw

All groups in the study will receive scheduled email and text message reminders to prompt the completion of assessment questionnaires at each time point (baseline and 3, 6, and 12 months). Emails will be sent 3, 6, and 9 days following the commencement of each phase, and text messages will be sent on day 9. Follow-up phone calls may also be scheduled at follow-up time points to prompt the completion of questionnaires. As an incentive to complete questionnaires and to promote retention, participants will automatically be entered into a gift voucher prize draw. Each draw will have a 1 in 100 chance of a prize, with the value of the gift voucher increasing in value over time (from Aus $100 [US $66.10] and up to Aus $400 [US $264.40] at 12 months).

### Sample Size

The sample size calculation was based on detecting changes in the primary outcome of diet quality score (AFRS), with adequate power to assess differences in daily servings of fruit and vegetables. The study aims for a between-group increase in ARFS of 2.2 (baseline SD 9.6) and fruit and vegetables serves per day of 0.56 (baseline SD 2.4) compared with no change in the control group [53]. To detect this between-group AFRS difference with an alpha of .05 and 80% power, 300 participants per arm across 3 arms are required, totaling 900 participants. Given that we also wish to examine this effect a priori in male and female subgroups separately, we will require 900 participants of each gender or 1800 in total. To allow for 10% loss to follow-up at 3 months, 20% at 6 months, and 30% at 12 months, the study requires a total sample of 2570 (1285 males and females each).

### Statistical Analysis

The outcome effects will first be evaluated using independent *t* tests, followed by repeated measures within-group changes in the ARFS diet quality score and modeling between-group changes over time using the generalized linear mixed model. The model will be fitted with ARFS at all follow-up points as the outcome variable, with fixed effects for group, time, baseline ARFS, and time by group interaction. Covariates, including PA, sleep, smoking, and social desirability, will be included in the statistical model as potential confounders. Statistical significance of the primary efficacy analysis will be based on Hochberg multiple testing procedures with a family-wise error rate for each time point held at 2.5%. The main analysis will use a generalized linear mixed model with the outcome at all time points and intention-to-treat principle. Sensitivity analyses will be conducted with the last observation carried forward, with multiple imputations, and for completers only. Analyses will be performed using SAS version 9.4 or later (SAS Institute Inc). All variables will be checked for plausibility and missing values. Data will be presented as mean (SD) for continuous and count variables.

### Health Economic Analysis

The economic analysis will be conducted from a health service perspective. Cost estimation will follow a categorize:quantify:value approach. In the absence of health service implications, the costs will reflect the resources required to generate, implement, and deliver the respective interventions. The valuation will be founded on the concept of opportunity cost, that is, the value of the benefit forgone in not employing labor, services, or materials in alternative uses. Market prices will be used as a proxy for this value. Labor costs will reflect relevant skills, such as dietician time and administration time, and will incorporate additional employee benefits such as superannuation. Services include expenditure such as Facebook advertising. Materials capture nonlabor cost items such as flyers. Costs will be reported separately and jointly.

If a statistically significant difference in AQoL-6D is found, a within-trial cost analysis will be conducted using quality-adjusted life years (QALYs) as the primary outcome. The incremental cost-effectiveness ratio (ICER) will report an incremental cost per QALY, reflecting the incremental outcome and cost differences between the comparator groups. The ICERs will be calculated as the arithmetic mean difference in cost between the intervention and control arm divided by the arithmetic mean difference in effect. Groups 2 and 3 will be compared individually with Group 1 and each other. If a significant change in AQoL-6D is not observed, the ARFS outcome measure will be used. If neither measure realizes a statistically significant change, the economic method will default to a cost-consequence analysis, with incremental costs reported against all primary and secondary outcomes. The economic analysis will be conducted, and the results will be reported in accordance with best practice guidelines [54,55].

## Results

Data collection commenced in February 2018 and is ongoing. As of December 2019, 1277 participants have been randomized.

## Discussion

### Principal Findings

The aim of this study is to evaluate the efficacy of delivering varying levels of personalized dietary feedback and support on improving the diet quality of young adults. Young adulthood is a period of major transition whereby changes during this period influence diet and eating behaviors that contribute to the weight gain trajectory that is common in this age group. Changes include social influences, changes to the home and school/work environment, and changes in financial circumstances, which add additional stresses during this period. The perceived effort, cost, peer influence, lack of time, and feelings of inferiority are barriers to making positive changes in eating patterns and other health-related behaviors [17]. Further complexity is added when we start to consider the many other layers that shape a person’s eating behaviors, including the food and beverage industry, access to health care, education, and social and cultural norms, and it needs to be acknowledged that active engagement from various segments of society is required. Approaches need to be incorporated into existing organizational structures to influence change at the population level.

### Strengths and Limitations

This protocol has been designed to address some of the major challenges related to improving dietary patterns of young adults, including the ease of access to personalized nutrition advice, education on cooking skills, and practical nutrition strategies such as how to eat on a budget and goal setting and tracking to ensure dietary changes remain realistic and achievable. What is novel is the use of validated web-based dietary assessment tools to connect young adults with personalized real-time feedback on their dietary intake, an online library of resources about healthy eating, goal setting, and access to a health professional.

### Conclusions

The results of this study will strengthen the current evidence related to improving nutrition by using technology-driven tools to address common barriers and motivators related to healthy eating and accessing personal dietary advice and support in young adults. The findings from testing efficacy and cost-effectiveness will inform approaches to reach and engage young adults. These will have major implications for future design and conduct of programs that target improved health and well-being in young adults.

## Appendix

Multimedia Appendix 1 Web interface.

Multimedia Appendix 2 Summary of Web-based measures and timing of data collection.

Multimedia Appendix 3 CONSORT-eHEALTH checklist (V 1.6.1).

## Abbreviations

AES: Australian Eating Survey
Aim4Me: Advice, Ideas, and Motivation for My Eating
APD: accredited practicing dietitian
AQoL-6D: 6-dimensional Assessment of Quality of Life scale
ARFS: Australian Recommended Food Score
COM-B: capability, opportunity, and motivation
FFQ: Food Frequency Questionnaire
HEQ: Healthy Eating Quiz
ICER: incremental cost-effectiveness ratio
NHMRC: National Health and Medical Research Council
PA: physical activity
PNQ: Personalized Nutrition Questionnaire
PNT: Personalized Nutrition Toolbox
QALY: quality-adjusted life year
SDT: self-determination theory
SSB: sugar-sweetened beverage

## References

[1] Allman-Farinelli M (2015). Nutrition promotion to prevent obesity in young adults. Healthcare (Basel).

[2] Munt AE, Partridge SR, Allman-Farinelli M (2017). The barriers and enablers of healthy eating among young adults: a missing piece of the obesity puzzle: A scoping review. Obes Rev.

[3] (2015). Australian Bureau of Statitics.

[4] Gomersall SR, Dobson AJ, Brown WJ (2014). Weight gain, overweight, and obesity: determinants and health outcomes from the Australian longitudinal study on women's health. Curr Obes Rep.

[5] Truesdale KP, Stevens J, Lewis CE, Schreiner PJ, Loria CM, Cai J (2006). Changes in risk factors for cardiovascular disease by baseline weight status in young adults who maintain or gain weight over 15 years: the CARDIA study. Int J Obes (Lond).

[6] Williamson DF, Kahn HS, Remington PL, Anda RF (1990). The 10-year incidence of overweight and major weight gain in US adults. Arch Intern Med.

[7] Bray GA (2004). Medical consequences of obesity. J Clin Endocrinol Metab.

[8] Nour MM, McGeechan K, Wong AT, Partridge SR, Balestracci K, Roy R, Hebden L, Allman-Farinelli M (2015). Diet quality of young adults enrolling in TXT2BFiT, a mobile phone-based healthy lifestyle intervention. JMIR Res Protoc.

[9] (2014). Australian Bureau of Statistics.

[10] Micha R, Khatibzadeh S, Shi P, Andrews K, Engell R, Mozaffarian D, Global Burden of Diseases Nutrition and Chronic Diseases Expert Group (NutriCoDE) (2015). Global, regional and national consumption of major food groups in 1990 and 2010: a systematic analysis including 266 country-specific nutrition surveys worldwide. BMJ Open.

[11] Imamura F, Micha R, Khatibzadeh S, Fahimi S, Shi P, Powles J, Mozaffarian D, Global Burden of Diseases Nutrition and Chronic Diseases Expert Group (NutriCoDE) (2015). Dietary quality among men and women in 187 countries in 1990 and 2010: a systematic assessment. Lancet Glob Health.

[12] Marshall S, Burrows T, Collins CE (2014). Systematic review of diet quality indices and their associations with health-related outcomes in children and adolescents. J Hum Nutr Diet.

[13] Wirt A, Collins CE (2009). Diet quality--what is it and does it matter?. Public Health Nutr.

[14] Jacka FN, Kremer PJ, Berk M, de Silva-Sanigorski AM, Moodie M, Leslie ER, Pasco JA, Swinburn BA (2011). A prospective study of diet quality and mental health in adolescents. PLoS One.

[15] Niccoli T, Partridge L (2012). Ageing as a risk factor for disease. Curr Biol.

[16] Liu K, Daviglus ML, Loria CM, Colangelo LA, Spring B, Moller AC, Lloyd-Jones DM (2012). Healthy lifestyle through young adulthood and the presence of low cardiovascular disease risk profile in middle age: the Coronary Artery Risk Development in (Young) Adults (CARDIA) study. Circulation.

[17] Ashton LM, Hutchesson MJ, Rollo ME, Morgan PJ, Thompson DI, Collins CE (2015). Young adult males' motivators and perceived barriers towards eating healthily and being active: a qualitative study. Int J Behav Nutr Phys Act.

[18] Holley TJ, Collins CE, Morgan PJ, Callister R, Hutchesson MJ (2016). Weight expectations, motivations for weight change and perceived factors influencing weight management in young Australian women: a cross-sectional study. Public Health Nutr.

[19] Ryan RM, Deci EL (2000). Self-determination theory and the facilitation of intrinsic motivation, social development, and well-being. Am Psychol.

[20] Michie S, van Stralen MM, West R (2011). The behaviour change wheel: a new method for characterising and designing behaviour change interventions. Implement Sci.

[21] Ashton LM, Sharkey T, Whatnall MC, Williams RL, Bezzina A, Aguiar EJ, Collins CE, Hutchesson MJ (2019). Effectiveness of interventions and behaviour change techniques for improving dietary intake in young adults: a systematic review and meta-analysis of RCTs. Nutrients.

[22] Michie S, Johnston M, Francis J, Hardeman W, Eccles M (2008). From theory to intervention: mapping theoretically derived behavioural determinants to behaviour change techniques. Appl Psychol.

[23] Schulz K, Altman D, Moher D, CONSORT Group (2010). CONSORT 2010 Statement: updated guidelines for reporting parallel group randomised trials. Trials.

[24] Whitaker C, Stevelink S, Fear N (2017). The use of Facebook in recruiting participants for health research purposes: a systematic review. J Med Internet Res.

[25] Australian Government Department of Health.

[26] Collins C The Healthy Eating Quiz.

[27] Collins CE, Burrows TL, Rollo ME, Boggess MM, Watson JF, Guest M, Duncanson K, Pezdirc K, Hutchesson M (2015). The comparative validity and reproducibility of a diet quality index for adults: the Australian Recommended Food Score. Nutrients.

[28] Williams R, Rollo M, Schumacher T, Collins C (2017). Diet quality scores of australian adults who have completed the healthy eating quiz. Nutrients.

[29] Collins CE, Boggess MM, Watson JF, Guest M, Duncanson K, Pezdirc K, Rollo M, Hutchesson MJ, Burrows TL (2014). Reproducibility and comparative validity of a food frequency questionnaire for Australian adults. Clin Nutr.

[30] National Health and Medical Research Council (2006). Nutrient Reference Values.

[31] Brain K, Burrows TL, Rollo ME, Hayes C, Hodson FJ, Collins CE (2019). The effect of a pilot dietary intervention on pain outcomes in patients attending a tertiary pain service. Nutrients.

[32] Eat for Health.

[33] Ashton L, Williams R, Wood L, Schumacher T, Burrows T, Rollo M, Pezdirc K, Callister R, Collins C (2017). Comparison of australian recommended food score (ARFS) and plasma carotenoid concentrations: A validation study in adults. Nutrients.

[34] Watson JF, Collins CE, Sibbritt DW, Dibley MJ, Garg ML (2009). Reproducibility and comparative validity of a food frequency questionnaire for Australian children and adolescents. Int J Behav Nutr Phys Act.

[35] Bush K, Kivlahan DR, McDonell MB, Fihn SD, Bradley KA (1998). The AUDIT alcohol consumption questions (AUDIT-C): an effective brief screening test for problem drinking. Ambulatory Care Quality Improvement Project (ACQUIP). Alcohol Use Disorders Identification Test. Arch Intern Med.

[36] Pursey K, Burrows TL, Stanwell P, Collins CE (2014). How accurate is web-based self-reported height, weight, and body mass index in young adults?. J Med Internet Res.

[37] Moodie M, Richardson J, Rankin B, Iezzi A, Sinha K (2010). Predicting time trade-off health state valuations of adolescents in four Pacific countries using the Assessment of Quality-of-Life (AQoL-6D) instrument. Value Health.

[38] Pelletier LG, Dion SC, Slovinec-D'Angelo M, Reid R (2004). Why do you regulate what you eat? Relationships between forms of regulation, eating behaviors, sustained dietary behavior change, and psychological adjustment. Motiv Emot.

[39] Gardner B, Abraham C, Lally P, de Bruijn G (2012). Towards parsimony in habit measurement: testing the convergent and predictive validity of an automaticity subscale of the Self-Report Habit Index. Int J Behav Nutr Phys Act.

[40] Deci EL, Ryan RM (2002). Handbook of Self-Determination Research.

[41] Zimet GD, Powell SS, Farley GK, Werkman S, Berkoff KA (1990). Psychometric characteristics of the Multidimensional Scale of Perceived Social Support. J Pers Assess.

[42] Godin G, Shephard RJ (1985). A simple method to assess exercise behavior in the community. Can J Appl Sport Sci.

[43] Marshall A, Miller Y, Burton N, Brown W (2010). Measuring total and domain-specific sitting: a study of reliability and validity. Med Sci Sports Exerc.

[44] Johns M (1991). A new method for measuring daytime sleepiness: the Epworth sleepiness scale. Sleep.

[45] Mullins R, Borland R (1998). CiteSeerX.

[46] Hughes JR, Keely JP, Niaura RS, Ossip-Klein DJ, Richmond RL, Swan GE (2003). Measures of abstinence in clinical trials: issues and recommendations. Nicotine Tob Res.

[47] Lovibond S, Lovibond P (1995). Manual for the Depression Anxiety Stress Scales. Second Edition.

[48] Hébert JR (2016). Social desirability trait: Biaser or driver of self-reported dietary intake?. J Acad Nutr Diet.

[49] Reynolds WM (1982). Development of reliable and valid short forms of the Marlowe-Crowne Social Desirability Scale. J Clin Psychol.

[50] Martin HJ (1984). A revised measure of approval motivation and its relationship to social desirability. J Pers Assess.

[51] Spanos S, Vartanian LR, Herman CP, Polivy J (2015). Personality, perceived appropriateness, and acknowledgement of social influences on food intake. Pers Individ Dif.

[52] Bitly Inc.

[53] O'Brien KM, Hutchesson MJ, Jensen M, Morgan P, Callister R, Collins CE (2014). Participants in an online weight loss program can improve diet quality during weight loss: a randomized controlled trial. Nutr J.

[54] Husereau D, Drummond M, Petrou S, Carswell C, Moher D, Greenberg D, Augustovski F, Briggs AH, Mauskopf J, Loder E, ISPOR Health Economic Evaluation Publication Guidelines-CHEERS Good Reporting Practices Task Force (2013). Consolidated Health Economic Evaluation Reporting Standards (CHEERS)--explanation and elaboration: a report of the ISPOR Health Economic Evaluation Publication Guidelines Good Reporting Practices Task Force. Value Health.

[55] Gerard K, Lowin A, Schuffham P, Haywood P, Hall J (2001). How to Compare the Costs and Benefits: Evaluation of the Economic Evidence.

