# Supplementary material for: Investigating the Efficacy and Cost-Effectiveness of Technology-Delivered Personalized Feedback on Dietary Patterns in Young Australian Adults in the Advice, Ideas, and Motivation for My Eating (Aim4Me) Study: Protocol for a Randomized Controlled Trial
Source: JMIR Res Protoc. 2020 May 22;9(5):e15999. doi: 10.2196/15999 (PMC7275255; doi:10.2196/15999)
Supplement: Multimedia Appendix 1 [file resprot_v9i5e15999_app1.pdf]

Supplementary material: Web interface

Figure 1: Dashboard

From the dashboard, participants can access their goals, personalised feedback reports, dietitian video consultations and educational content.

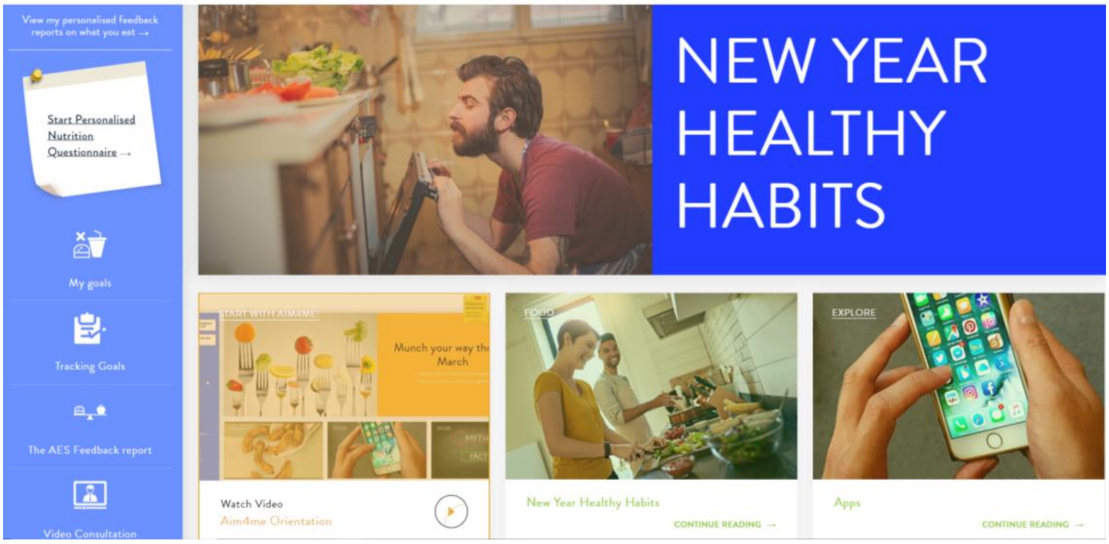

Figure 2: AES Feedback report

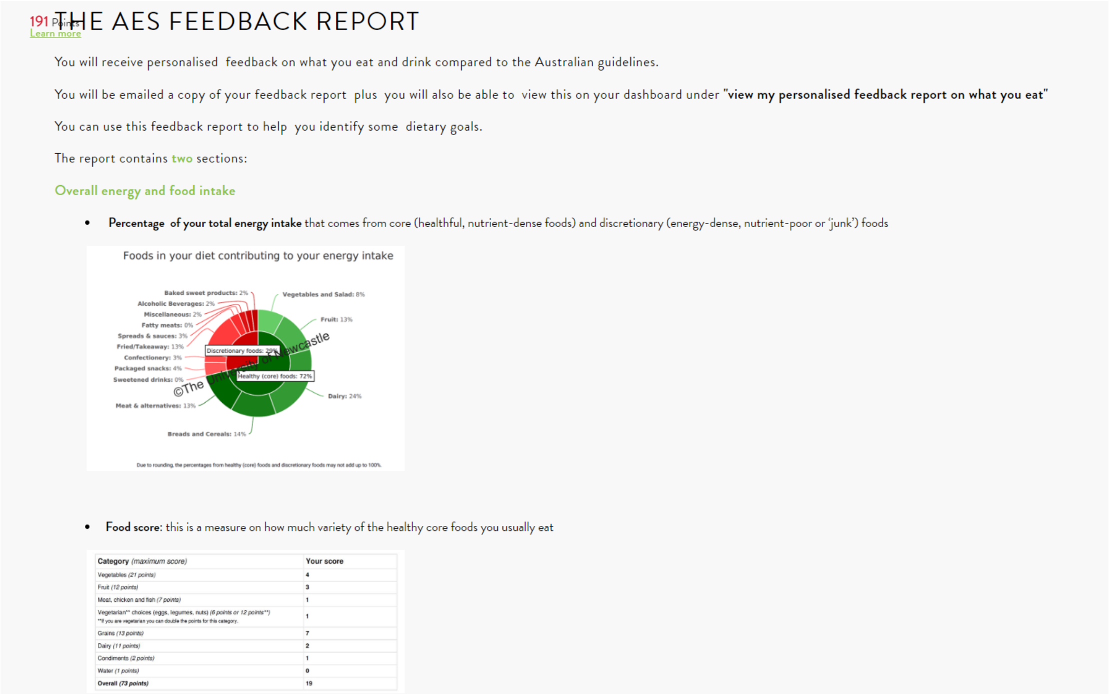
191 Points
Learn more

THE AES FEEDBACK REPORT

You will receive personalised feedback on what you eat and drink compared to the Australian guidelines.

You will be emailed a copy of your feedback report plus you will also be able to view this on your dashboard under **"view my personalised feedback report on what you eat"**

You can use this feedback report to help you identify some dietary goals.

The report contains two sections:

Overall energy and food intake

- **Percentage of your total energy intake** that comes from core (healthful, nutrient-dense foods) and discretionary (energy-dense, nutrient-poor or 'junk') foods

- **Food score:** this is a measure on how much variety of the healthy core foods you usually eat

**Category** *(maximum score)*	**Your score**
Vegetables *(21 points)*	**4**
Fruit *(12 points)*	**3**
Meat, chicken and fish *(7 points)*	**1**
Vegetarian** choices (eggs, legumes, nuts) *(6 points or 12 points**)*<br>**If you are vegetarian you can double the points for this category.	**1**
Grains *(13 points)*	**7**
Dairy *(11 points)*	**2**
Condiments *(2 points)*	**1**
Water *(1 points)*	**0**
**Overall *(73 points)***	19

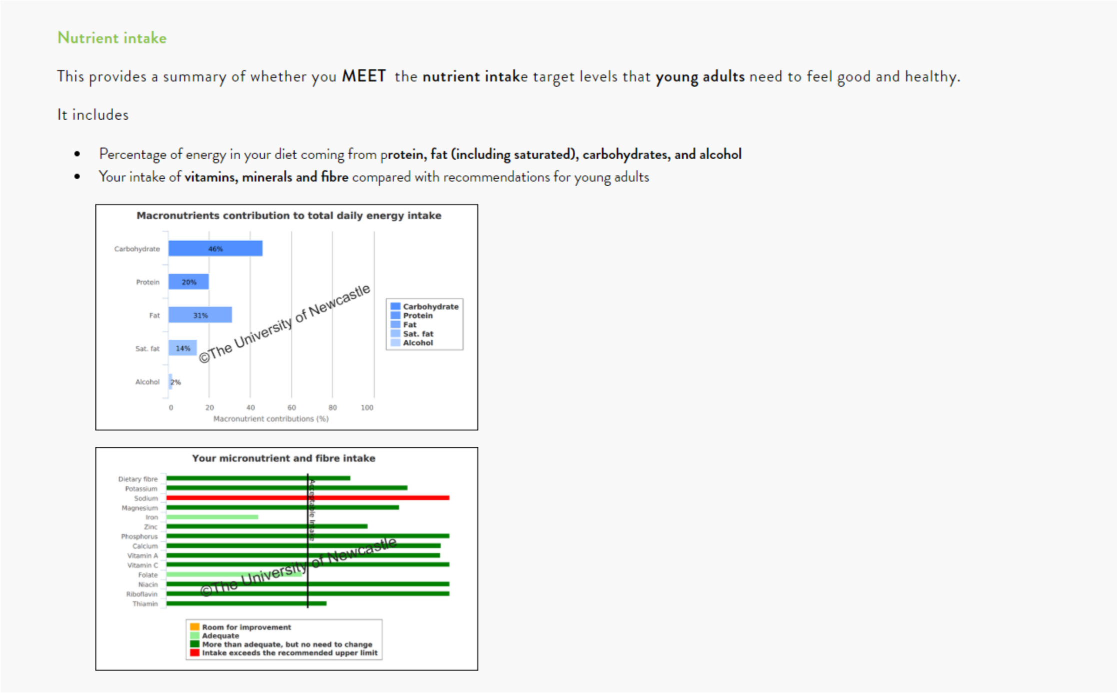

Figure 3: Goal setting feature

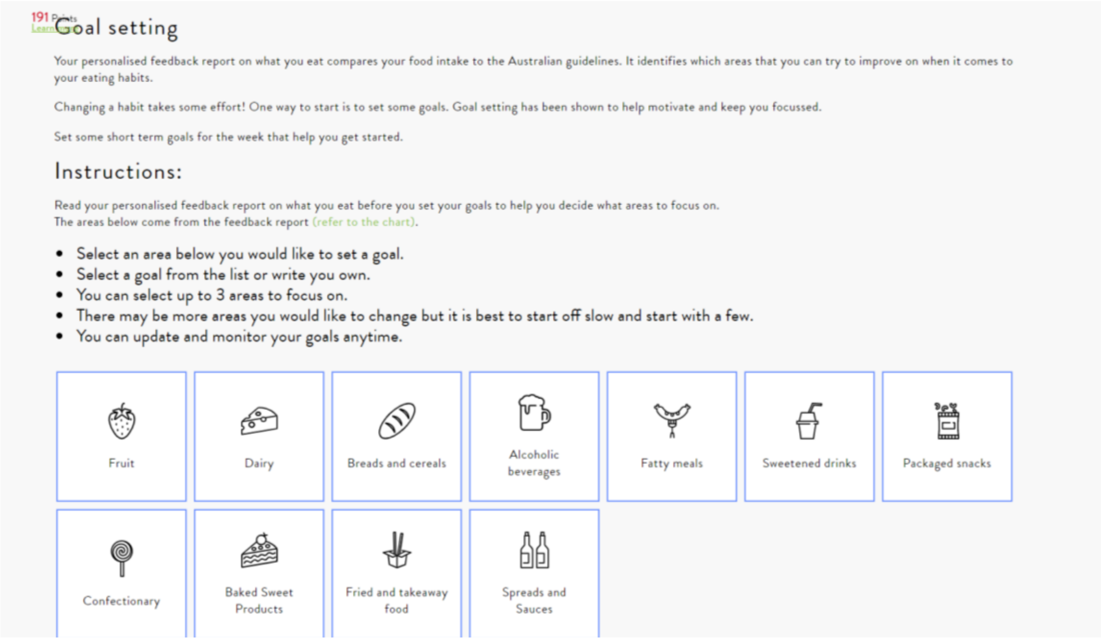

Figure 4: Goal tracking feature

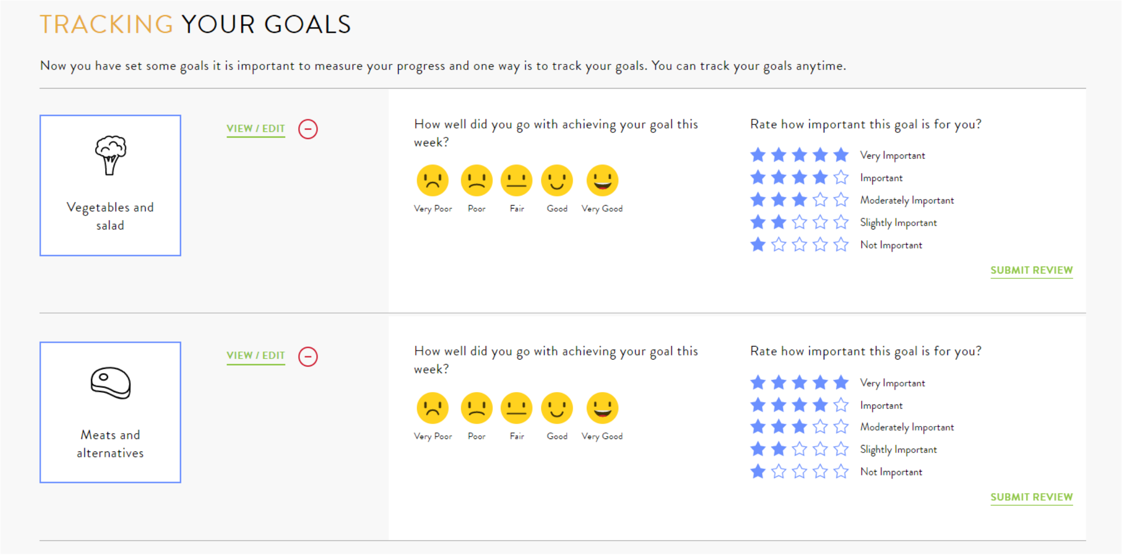

Figure 5: Task bar

The task bar allows participants to navigate through the education content. This includes the “Theme of the Month” which provides education and advice on various nutrition topics and covers key occasions across the year such as New Year habits and Dry July.

Participants can also access the “Food” and “Explore” tabs which provide additional information and resources such as recipes, cooking tips, how to read food labels and navigate grocery shops and provides guidance on useful apps for helping to improve dietary intake.

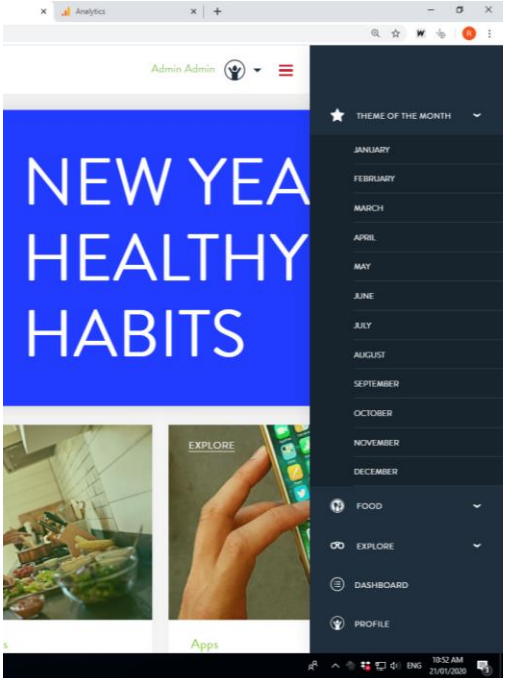

Plan your food shopping 07 Jan 2020

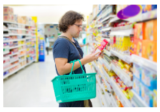

Planning your food shopping is a great way to ensure that you are making healthy, nutritious choices, and also sticking to a pre-panned budget. Using a weekly planner, making a shopping list, checking...

CONTINUE READING

Be a savvy shopper 07 Jan 2020

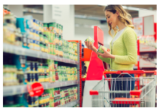

8 tips for becoming a savvy shopper:•Check supermarket catalogues or browse online for deals and specials and identify any products that you need before going shopping •Visit your local...

CONTINUE READING

Reduce luxury food purchases 07 Jan 2020

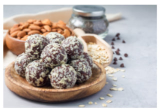

Swapping (not stopping) some of you grocery purchases is a great way to save money each week. 'Luxury' food purchases can add up very quickly and soon blow out your food budget. Top tips so save...

CONTINUE READING

Cut down on waste 07 Jan 2020

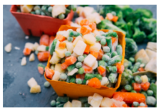

Food waste is a huge issue worldwide. The Food and Agricultural Organization estimated that more than one billion tonnes of food is wasted worldwide every year. In Australia, this is around 4.2 million...

CONTINUE READING

FRUIT AND VEGETABLES

Eat more fruit and veg- what's in it for you? 01 Mar 2018

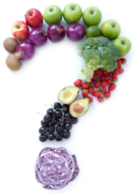

You've heard it before fruits and veggies are good for you. Here are some TOP reasons why you should add more fruit and veggies.

CONTINUE READING

Eat a rainbow everyday 01 Mar 2018

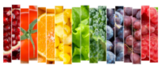

It's important that you eat the right amount of fruit and veggie serves per day but it's ALSO about eating a wide VARIETY so your body gets all the essential nutrients it needs!

CONTINUE READING

Eating 2 serves of fruit and 5-6 serves of veggies is POSSIBLE !

Vegetables just don't taste good! 01 Mar 2018

RECIPES

Vegetarian Recipes 01 Mar 2018

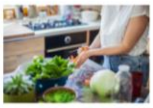

Here are some quick and easy vegetarian recipes to kick start you for Meatless Monday.

CONTINUE READING

Healthy gut recipes 01 Jun 2018

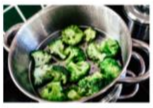

CONTINUE READING

Recipes to fuel your exercise 01 Aug 2018

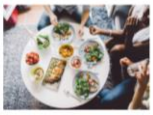

The Australian Institute of Sport have developed a range of recipes to help you fuel and recover from exercise. Each recipe provides the nutrient breakdown so you can check the protein and carbs in each...

CONTINUE READING

Quick and simple recipes 12 Sep 2018

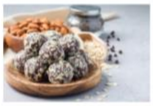

...

CONTINUE READING
