# Supplementary material for: Investigating the Efficacy and Cost-Effectiveness of Technology-Delivered Personalized Feedback on Dietary Patterns in Young Australian Adults in the Advice, Ideas, and Motivation for My Eating (Aim4Me) Study: Protocol for a Randomized Controlled Trial
Source: JMIR Res Protoc. 2020 May 22;9(5):e15999. doi: 10.2196/15999 (PMC7275255; doi:10.2196/15999)
Supplement: Multimedia Appendix 2 [file resprot_v9i5e15999_app2.docx]

| **Outcome** | | **Measure and number of items** | **Assessment time point** |
| --- | --- | --- | --- |
| **Primary outcomes** | | | |
|  | **Diet quality** | **Australian Recommended Food Score, 70 items [27]** | **Baseline** and 3, 6, and 1**2 months** |
| **Secondary outcomes** | | | |
|  | **Dietary intake** | **Australian Eating Survey food frequency questionnaire, 120 items^a^ [29]** | **Baseline** and 3, 6, and **12 months** |
|  | **Alcohol intake** | Alcohol Use Disorders Identification Test-Consumption, 3 items [35] | **Baseline and 3, 6, and 12 months** |
|  | **Anthropometrics and BMI** | **Self-report height and weight, 2 items** | **Baseline and 3, 6, and 12 months** |
|  | **Quality of life** | **AQOL-6D^b^, 21 items [37]; used in economic evaluations** | **Baseline and 3, 6, and 12 months** |
| **Covariates** | | | |
|  | **Social influences** | **Social Eating Scale, 6 items [51]; social desirability, 33 items [48,49]** | **Baseline and 3, 6, and 12 months** |
|  | Self-reported physical activity, sitting time, and sleep | **Godin Leisure-Time Exercise Questionnaire, 7 items [42]; Marshal Sitting Time Questionnaire, 10 items [43]; and Epworth Sleepiness Scale, 8 items [44]** | **Baseline and 3, 6, and 12 months** |
| **Smoking** | | **Smoking 7-day abstinence, 2 items [46]** | **3, 6, and 12 months** |
| **Depression anxiety and stress** | | **Depression, Anxiety and Stress Scale, 21 items [47]** | **Baseline and 3, 6, and 12 months** |
| **Self-determination factors** | | **Regulation of Eating Behavior Scale, 24 items [38]; Self-Report Behavioral Automaticity Index, 4 items [39]; Perceived Competence Scale, 4 items [40]; and Perceived Social Support Scale, 12 items [41]** | **Baseline and 3, 6, and 12 months** |
| **Economic analysis** | | | |
|  | | Australian Recommended Food Score or AQoL-6D | **Baseline and 3, 6, and 12 months** |
|  | | Cost estimates: costs relating to the intervention and implementation of the intervention, including materials, labor, and other expenditures | **Continuous** |
| **Engagement** | | **Website database, log-ins, visits to Web pages, viewed/visited personalized dietary feedback, viewed and completed goal setting and tracking, attendance at video consultation (Group 2), and Google Analytics (eg,** time of day access, device used, and number and duration of sessions) | **Continuous** |
| Reach | | **Google Analytics (eg, geographical location and referral sources) and Bitly links on recruitment materials** | **During recruitment** |
| Recruitment success | | **Recruitment strategies, expression of interest, number screened for eligibility, number consented, and number randomized** | **Continuous throughout recruitment period.** |
| Sociodemographic | | **Commonly used items drawn from national surveys** | **Baseline and 3, 6, and 12 months** |
| Satisfaction | | **Self-reported on intervention components and overall intervention** | **3, 6, and 12 months** |

**^a^A total of 120 diet-related questions are included in the** Australian Eating Survey**. This includes 71 diet-related questions that also make up the** Australian Recommended Food Score**.**

**^b^**AQoL-6D: 6-dimensional Assessment of Quality of Life.
